# Changes in the sagittal pharyngeal airway dimension following levelling and alignment in class II division 2 patients: a prospective clinical trial

**DOI:** 10.1007/s00784-026-06944-2

**Published:** 2026-06-05

**Authors:** Farah Y. Eid, Ahmed M. Madian, Dina Elfouly

**Affiliations:** Department of Orthodontics, Faculty of Dentistry, Alexandria University, Alexandria, Egypt

**Keywords:** Class II Division 2, Sagittal pharyngeal airway dimension, Mandibular position, Lateral cephalometric x-ray

## Abstract

**Objectives:**

This study aimed to evaluate changes in the sagittal pharyngeal airway dimensions (SPAD) and the related mandibular position following maxillary levelling and alignment in late adolescent Class II division 2 (Class II/2) patients.

**Materials and methods:**

Thirty Class II/2 patients (mean age 14.07 ± 1.21 years) were included. Pre-treatment orthodontic records were acquired, including panoramic and lateral cephalometric x-rays (**T0)**, photographs, and study models, followed by the onset of levelling and alignment in the upper arch. Lateral cephalometric x-rays and photographs were repeated six months later at the termination of this stage (**T1**), and measurements were performed in comparison to T0. SPAD was assessed at the nasopharyngeal, oropharyngeal, and laryngopharyngeal levels. Mandibular position was evaluated using the Ba–Ar distance, while sagittal and vertical skeletal relationships were assessed using SNA°, SNB°, ANB°, and FMA°. Upper incisor inclination was accounted for using the U1-FH**°** measurement.

**Results:**

Alignment of the maxillary arch in Class II/2 patients elicited a significant SPAD increase at the nasopharyngeal and the oropharyngeal levels (*p* < 0.001), with a non-significant change evoked at the laryngopharyngeal level (*p* = 0.42). Antero-posteriorly, a significant increase in the SNAº, SNBº, together with a significant decrease in the ANBº angle have been documented at T1. A significant increase in the Ba-Ar has been noted at T1 in contrast to T0, in addition to a significant reduction in the vertical dimension (FMAº). Dentally, a significant increase in the U1-FHº has been documented following the implemented treatment stage (*p* < 0.001).

**Conclusions:**

Results of this study may suggest that maxillary levelling and alignment in Class II/2 patients during the decelerating growth phase is associated with augmentation of the nasopharyngeal and oropharyngeal airways. These changes coincide with the significant labial tipping of the maxillary incisors and subsequent functional mandibular advancement, which collectively improve the intermaxillary relationship and reduce the vertical skeletal dimension. While natural growth cannot be entirely excluded, the rapid change suggests that these improvements are closely linked to the functional mandibular response.

**Clinical Relevance:**

Maxillary alignment in Class II division 2 cases may enhance upper airway patency by triggering a forward functional mandibular response, highlighting a potential link between orthodontic correction and upper airway patency.

Name of the Registry: ClinicalTrials.gov

Trial Registration Number: NCT06602518

Date of Registration: 19/09/2024 “Retrospectively registered”.

URL: https://clinicaltrials.gov/study/NCT06602518?cond=nct06602518&rank=1

## Background

Class II division 2 (Class II/2) is a distinctive subclass of Class II malocclusion, represented by unique dental and craniofacial features that distinguish it from other forms of malocclusion. These features include excessively retroclined maxillary incisors, deep overbite, and minimal overjet [1, 2]. Moreover, the anterior relationship displayed in Class II/2 cases has been presumed to generate posterior and superior condylar displacement in the glenoid fossa, and a subsequent posterior mandibular shift [3, 4], a characteristic that has been reported in approximately 1/3 of Class II/2 cases [3]. While the exact cause of Class II/2 malocclusion is still a subject of debate, several theories attempt to explain its origin. Some researchers argue that the condition stems from a skeletal discrepancy, such as an underdeveloped mandible or a lower jaw positioned too far back relative to the cranial base [1, 5]. Conversely, other experts contend that the primary driver is dentoalveolar, suggesting that the issue lies within the positioning of the teeth and their supporting bone, rather than the overall jaw structure [6, 7].

The existence of “functional mandibular retrusion” in some cases, has been reported to aid in the correction of the Class II relationship [8], where the creation of a sufficient overjet could permit the mandible to move into a forward position achieving a normal centric relation. Rickets recommended “unlocking” the deep bite by advancing the maxillary incisors, which will subsequently simulate the presentation of a Class II division 1, where treatment will involve more dental changes than skeletal ones in growing subjects [9]. Accordingly, “freeing” the mandible whether by labial tipping of the maxillary incisors through levelling and alignment, or by disarticulating the mandible and allowing it to assume a normal position as dictated by the musculature through placing a bite plate, would be beneficial in the diagnosis of functional mandibular retrusion, if present [10]. However, despite the fact that posterior mandibular shift should not be considered a consistent finding in Class II/2 cases, clinicians’ awareness of this possibility is of utmost importance for adequate orthodontic management [10].

With the continuous developments in medical care, the pharyngeal airway space in orthodontic patients has caught the attention of many researchers and clinicians. Pharyngeal airway size is a factor of prime importance that is known to affect the quality of sleep the patient exhibits [11, 12]. Moreover, the size of the nasopharynx has a detrimental effect on the pattern of breathing, whether it is oral or nasal [13].

Amidst the array of craniofacial anomalies, skeletal Class II has been found to be continuously linked to narrowing of the upper airways [14, 15]. Reviewing the literature, there is a relative agreement that maxillary protrusion increases the length of the upper airway [16, 17], whereas mandibular retrusion is correlated with its constriction [14, 15, 18]. The pharyngeal airways can be separated into three distinct levels, namely the nasopharyngeal, the oropharyngeal, and the laryngopharyngeal levels. Among these three sectors, the oropharyngeal is the one most likely influenced by the size and position of the tongue, which is linked to the hyoid bone [19].

An existing knowledge gap stems from the fact that the impact of a subsequent change in mandibular position on the sagittal pharyngeal airway dimensions (SPAD) remains unstudied in Class II/2 patients during the decelerating stages of the adolescent growth. This is specifically relevant in Class II/2 patients after the maxillary arch has been levelled, aligned, and the incisors’ axial inclination corrected. Therefore, the objective of this study was to compare the changes in the SPAD before and after maxillary levelling and alignment and overjet correction in Class II/2 subjects. Moreover, changes in the mandibular position, sagittal and vertical skeletal changes, as well as dental changes were assessed. The null hypothesis was that there are no significant differences in the SPAD before and after maxillary levelling and alignment in Class II/2 subjects, in this particular age group.

## Materials and methods

### Study design and participants

Ethical approval was procured for this prospective clinical trial from the Institutional Review Board of the Faculty of Dentistry, Alexandria University, Alexandria, Egypt (IORG:0008839, Protocol no. 0924-05/2024). Subjects were recruited from the outpatient clinic of the Department of Orthodontics, where those selected were examined and screened, taking into consideration the following eligibility criteria: (1) Class II/2 subjects as per the British Standard Institute Classification [20]; (2) Age ranging from 12 to 16 years; (3) Cervical Vertebral Maturational Index (CVMI) at stages 4, and 5 [21]; (4) Contact between maxillary and mandibular incisors, with the presence of a deep bite of at least 70% [21]; (5) Full permanent dentition, aside from the third molars. Subjects were excluded from the sample if they had previous orthodontic treatment, history of craniofacial trauma, or exhibited chronic respiratory problems that may affect the pharyngeal airway space, severe transverse problems, pain and/or clicking sound in the temporomandibular joint (TMJ) [22], and finally, those with craniofacial anomalies or diagnosed syndromes.

The calculated sample size was based on 80% study power and 5% alpha error. Marzouk and El Kalza [23] reported mean (SD) pre- and post-levelling oropharyngeal airway volume = 7.24 mm³ ± 1.43 and 7.93 mm³ ± 1.51, respectively. Based on comparison of means, using paired samples t-test, the required sample size was calculated to be 27 patients, increased to 30 to make up for cases lost to follow up [24]. Sample size estimation was carried out using MedCalc Statistical Software version 19.0.5 (MedCalc Software bvba, Ostend, Belgium; https://www.medcalc.org; 2019).

## Patient preparation

The enrolled subjects were prepared for fixed orthodontic treatment by recording medical and dental histories, in addition to routine orthodontic records (intra-oral and extra-oral photographs, x-rays, and dental models). The acquired lateral cephalograms at this stage were considered the pre-treatment records (T0). Patients were also instructed to undergo full mouth scaling and polishing, followed by proper oral hygiene instructions (using toothbrush, dental floss, and interdental brush). This was preceded by a complete and through explanation regarding the study procedures to both the participants and their parents/guardians, and accordingly, a written informed consent was obtained from each enrolled subject.

## Orthodontic treatment

All the enrolled patients underwent conventional fixed appliance therapy by bonding straight wire Roth appliances (0.022x0.028-in bracket slot) in the maxillary arch. The bonding procedure was standardized in all patients and performed by the same operator (F.Y.) using light-cured composite.

**Fig. 1:**
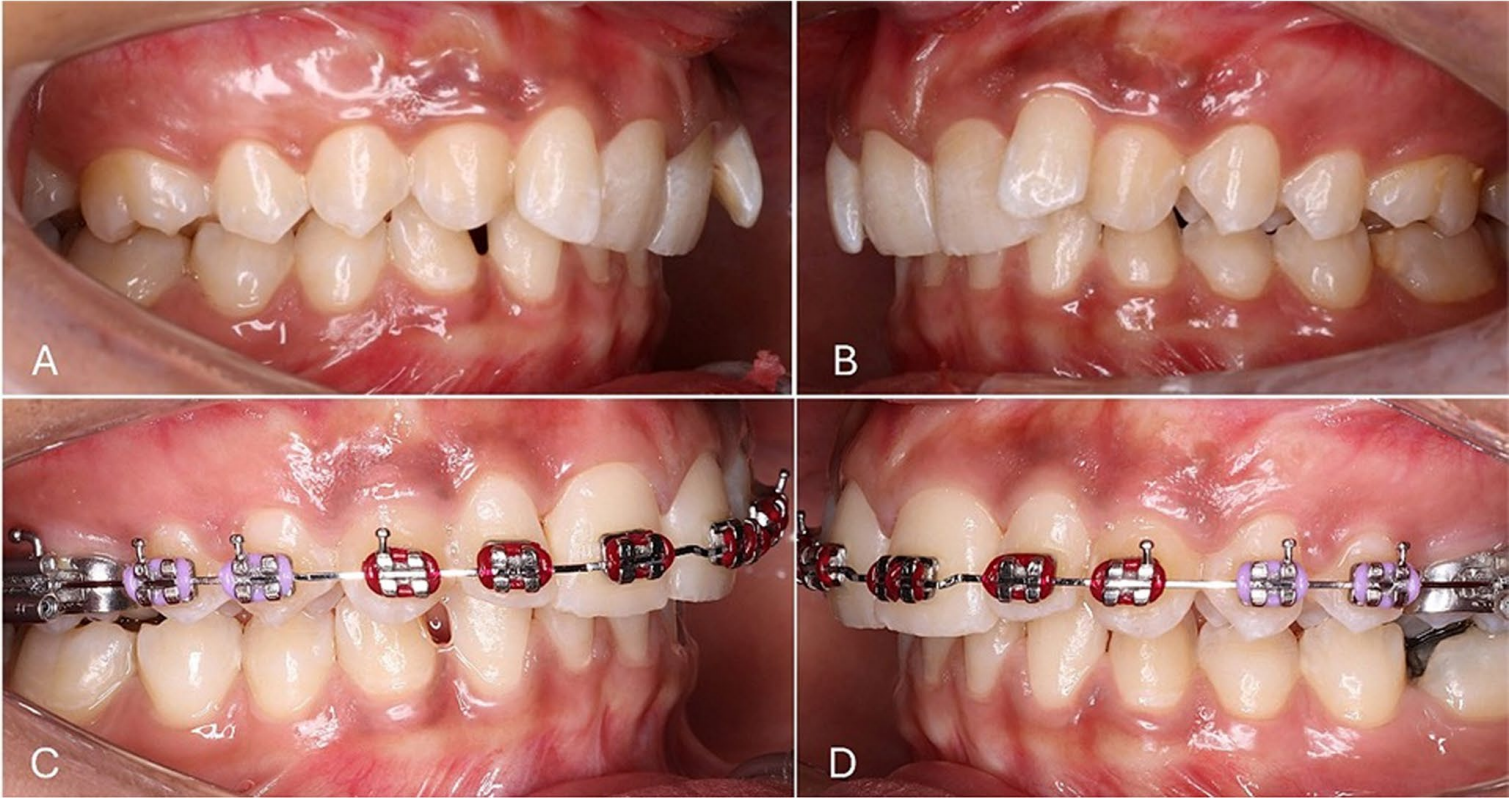
(**A**) Right, and (**B**) left pre-treatment intra-oral photographs (T0). (**C**) Right, and (**D**) left intra-oral photographs at T1, after levelling and alignment of the maxillary arch, and 2 months of stabilization with a 0.019 × 0.025-in SS arch wire

Levelling and alignment commenced using the following wire sequence: 0.014-in nickel titanium (NiTi), 0.018-in NiTi, 0.017 × 0.025-in NiTi, and 0.019 × 0.025-in NiTi arch wires. The upper arch was then stabilized with a 0.019 × 0.025-in stainless steel (SS) arch wire in place for 2 months. Levelling and alignment together with stabilization required approximately 6 months of treatment (Fig. 1). Lateral cephalometric x-rays were taken after the completion of this stage (T1), following the achievement of a well-aligned maxillary arch. The lateral cephalograms were taken in a natural head position (NHP) at both T0 and T1.

## Outcomes’ measurement

The acquired pre- and post-levelling and alignment lateral cephalometric x-rays (T0, T1) were taken using Veraviewepocs 3D R100 (J Morita Corp, Koyoto, Japan), operating at 60-90 kV and 8 mA, with an exposure time of 5 s, a field of view (FOV) of 25 × 20 cm, and a voxel size of 96 μm pixel size (high-resolution Ceph imaging). Ear rods and Nasion support for stabilization were used during image acquisition cephalometric capture (not stitched), reducing distortion. The final cephalometric radiographs were digitally traced and measured using Osirix open-source software. This DICOM-based software was used to identify all landmarks and perform quantitative assessments of linear distances (mm), angles (º), and sagittal pharyngeal airway areas (mm²) [25]. Accordingly, various landmarks were identified, and the following outcomes were measured:

1. **SPAD**

**Fig. 2:**
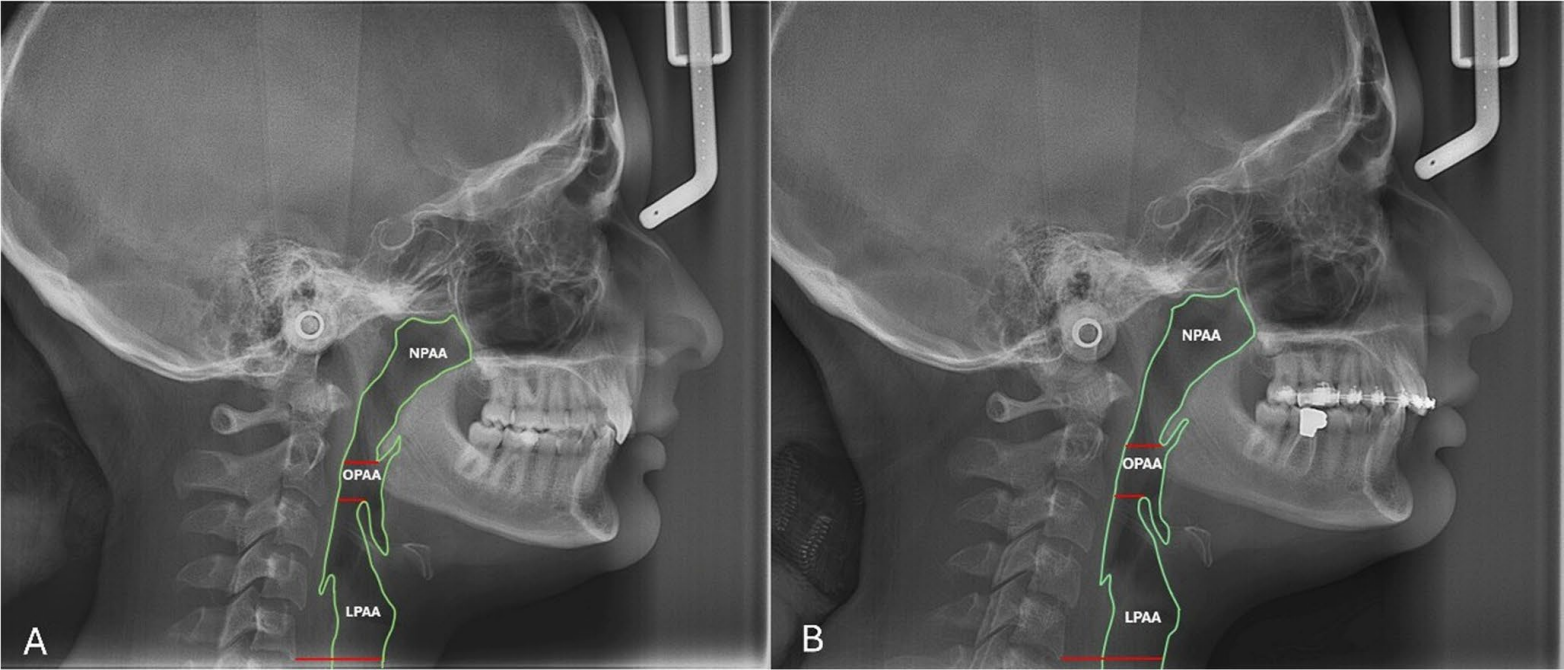
SPAD changes at the three tested levels; NPAA, OPAA, and LPAA, as measured from lateral cephalometric x-rays. **(A)** Pre-treatment at T0, and **(B)** Post-treatment at T1

The sagittal pharyngeal airway space was divided into the Nasopharyngeal airway area (NPAA), the Oropharyngeal airway area (OPAA), and the Laryngopharyngeal airway area (LPAA) [26]. The upper border of NPAA was delineated by a line extending from the Harmonium (H) to the posterior nasal spine (PNS). The lower extent of the NPAA was identified by drawing out a line extending from the tip of the soft palate parallel to the Frankfurt Horizontal plane (FH), to the posterior wall of the pharynx. The OPAA and LPAA were discerned by a line drawn at the level of the tip of epiglottis, parallel to the FH plane to the posterior wall of the pharynx. Another line that was parallel to FH plane, passing through the antero-inferior most point (C5AI) of the fifth cervical vertebra designated the inferior border of the LPAA (Fig. 2). The area was measured using the same software in mm².

2. **Changes in the mandibular position**

The distance between the Basion and Articulare points (Ba-Ar) was measured on the acquired lateral cephalograms at T0 and T1, and the difference between both measurements determined the amount of the forward mandibular shift generated after overjet correction, if present.

3. **Sagittal and vertical skeletal measurements**

Using the procured pre- and post-levelling and alignment lateral cephalograms, several linear and angular measurements were performed, where a sagittal as well as a vertical evaluation was conducted (Table 1). For assessment in the sagittal dimension, the angles SNA°, SNB°, and ANB° were measured. As for the vertical dimension, the Frankfurt-mandibular plane angle (FMA°) was assessed for evaluation.

**Table 1:** Skeletal and dental measurements; SNA°, SNB°, ANB°, FMA°, Ba-Ar (mm). U1-FHº

SNA°	Angle between the points Sella (S), Nasion (*N*) and A point, and shows the anteroposterior position of the maxilla relative to the anterior cranial base.
SNB°	Angle between points Sella (S), Nasion (N), and B point, and describes the anteroposterior position of the mandible relative to the anterior cranial base.
ANB°	Angle between points A, Nasion (N) and B point, indicating the skeletal relationship between the maxilla and the mandible.
FMA°	Angle between the Mandibular plane and the Frankfort horizontal plane.
Basion	The most anterior point on the margin of foramen magnum.
Articulare	The intersection of the shadow of the undersurface of the Basi-occiput with that of the posterior border of the neck of the mandible, with the teeth being in centric occlusion.
Ba-Ar (mm)	Linear distance between the Basion and Articulare points.
U1-FHº	Angle between the maxillary incisor’s long axis and the Frankfurt-horizontal plane.

4. **Dental measurements (upper incisor axial inclination)**

To assess dental inclination, the Upper incisor-Frankfurt horizontal plane angle (U1-FH°) was included as a measure of the upper incisors’ axial inclination via lateral cephalograms and compared across the T0–T1 interval (Table 1).

## Reliability testing

Initially, one researcher performed all measurements (D. E.). The same and another calibrated independent investigator (F. E.) repeated the whole measurements on 10 randomly selected x-rays, 2 weeks later to test intra and inter-examiner reliability using intraclass correlation coefficient (ICC) [27].

### Statistical analysis

Normality was tested for all variables using descriptive statistics, Q-Q plots, histograms, and Shapiro Wilk normality test. All variables showed normal distribution, so means and standard deviation (SD) were calculated, and parametric tests were used. Comparisons of skeletal and dental readings, as well as airway measurements at both T0 and T1 were performed using paired samples t-test with calculation of mean differences and 95% confidence intervals (CI)s. Significance level was set at p-value < 0.05. Data were analyzed using IBM SPSS for Windows (Version 26.0).

## Results

Baseline characteristics of the enrolled patients’ sample are summarized in Table 2. The average values (means) and variability (standard deviations) for all measured outcomes were determined at baseline (T0) and post-levelling and alignment of the maxillary arch (T1). The differences between both time points, representing the net change, are included in Tables 3 and 4. On another note, the calculated ICC values ranged from 0.81 to 0.88, indicating excellent intra-examiner and inter-examiner reliability [27].

**Table 2:** Sample description (n= 30)

Age in years	Mean (SD)	14.07 (1.21)
	Min – Max	12.25 – 16.00
Gender: n (%)	Male	13 (43.3%)
	Female	17 (56.7%)

**Table 3:** Comparison of airway area (mm²) measurements pre-treatment (T0) and post-treatment (T1)

	T0	T1	Difference (T1-T0)	95% CI	*P* value
	Mean (SD)				
NPAA	369.27 (10.23)	438.40 (14.05)	69.13 (9.47)	65.60, 72.69	< 0.001*
OPAA	177.30 (21.30)	215.93 (26.11)	38.63 (5.22)	36.69, 40.58	< 0.001*
LPAA	257.17 (28.89)	259.87 (30.02)	2.70 (2.31)	-3.99, 9.39	0.42

*SD* Standard Deviation, *CI* Confidence interval

Paired t-test was used

*statistically significant at p-value < 0.05

**Table 4:** Comparison of skeletal and dental readings between T0 and T1

	T0	T1	Difference (T1-T0)	95% CI	*P* value
	Mean (SD)				
SNAº	81.71 (1.71)	82.77 (1.63)	1.06 (0.49)	0.88, 1.24	< 0.001*
SNBº	74.63 (1.45)	76.93 (1.54)	2.29 (0.72)	2.02, 2.56	< 0.001*
ANBº	7.08 (1.51)	5.84 (1.53)	-1.23 (0.65)	-1.48, -0.99	< 0.001*
FMAº	27.81 (3.46)	25.45 (3.42)	-2.37 (0.41)	-2.52, -2.12	< 0.001*
Ba-Ar (mm)	6.13 (1.18)	6.70 (1.13)	0.57 (0.22)	0.48, 0.65	< 0.001*
U1-FHº	95.86 (1.87)	113.21 (2.63)	17.36 (2.40)	16.46, 18.25	< 0.001*

*SD* Standard Deviation, *CI* Confidence interval

Paired t-test was used

*statistically significant at p-value < 0.05

## Sagittal Pharyngeal airway dimension

Table 3 depicts the changes observed in the sagittal pharyngeal airway area across the three measured levels (NPAA, OPAA, and LPAA), at T1 in contrast to T0. Starting with NPAA level, a significant increase has been recorded with a value of 69.13 mm² ± 9.47 after levelling and alignment of the maxillary arch (*p* < 0.001). Moving to the OPAA level, and in alignment with the airway area increase in the NPAA, a significant increase of 38.63 mm² ± 5.22 was observed at T1 when compared to T0 (*p* < 0.001). For the final LPAA level, which is the lowest level assessed, a non-significant change (2.70 mm² ± 2.31) has taken place in the airway area of the tested subjects, when pre- and post-treatment values were compared (*p* = 0.42).

### Changes in mandibular position

Linear measurements representing the changes in mandibular position succeeding overjet establishment are delineated in Table 4. A statistically significant increase in the Ba-Ar distance has been evoked after unlocking the mandible (*p* < 0.05), denoting the forward displacement of the Articulare point by 0.57 mm ± 0.22.

### Sagittal and vertical skeletal measurements

Sagittal and vertical skeletal changes between T0 and T1 are represented in Table 4. For the sagittal measurements, levelling and alignment of the maxillary arch evoked a significant change in the SNA°, SNB° and ANB° values at T1. A statistically significant increase of 1.06° ± 0.49 in the SNAº, and an increase of 2.29° ± 0.72 in the SNB° values have been documented at T1 in comparison to those at T0 (*p* < 0.001). On the other hand, ANB° values showed a statistically significant decrease of -1.23° ± 0.65 post-treatment (*p* < 0.001), naturally reflecting the significant change in the intermaxillary relationships.

The recorded FMAº values at T1 as opposed to those at T0, unveiled a statistically significant decrease in the vertical dimension after the implemented treatment phase (*p* < 0.05), with a documented difference of -2.37° ± 0.41.

### Dental changes (upper incisor axial inclination)

Maxillary incisor inclination increased significantly from a baseline (T0) of 95.86º ± 1.87, to 113.21º ± 2.63 at T1 (*p* < 0.001). This reflects statistically significant labial tipping or proclination of upper incisors during the levelling and alignment stage.

## Discussion

The present study was conducted to test the influence of the probable forward mandibular displacement proceeding levelling and alignment of the maxillary arch in Class II/2 patients on the SPAD in the decelerating stages of growth. This goes back to reports suggesting that “functional mandibular retrusion” exists in such cases, correctable by ensuring adequate overjet to encourage forward movement of the mandible [8]. Based on the available evidence, this critical research point remains unexamined by a prospective clinical trial, affirming that a major gap in our clinical understanding persists. As per the reported outcomes, the null hypothesis has been mostly rejected, where a statistically significant increase in the SPAD has been documented at the NPAA, and the OPAA levels, whereas a non-significant change has been noted at the lowest level (LPAA).

With a mean age of 14.07 ± 1.21 years, the recruited subjects represent the late, or decelerating stage of the adolescent growth spurt. This age range (12–16 years) is relevant as research confirms that a significant nasopharyngeal growth deceleration begins approximately around the age of 13 years [28, 29], hence the aim of the present study as priorly stated.

The selection of lateral cephalograms for the quantitative assessment of SPAD changes between T0 and T1 was based on their availability as routine diagnostic radiographs [26], in addition to them being the tools of choice in several former investigations [30–32]. Moreover, they were preferred for their cost-effectiveness and diminished radiation dose compared to other imaging modalities, all while maintaining proven accuracy and reliability [33]. Researchers have also found a significant agreement between pharyngeal airway dimensions measured on lateral cephalometric radiographs and the corresponding volumetric data generated by three-dimensional (3D) Cone Beam Computed Tomography (CBCT) [34].

On another note, although linear metrics have been the exclusive method used by some researchers to quantify pharyngeal airway parameters, Aboudara et al. [35] demonstrated that sagittal airway area measurements exhibit a more statistically significant correlation with 3D volumetric changes compared to their linear counterparts. Therefore, the present investigation implemented sagittal pharyngeal airway area measurements in methodological accordance with several preceding studies [31, 32, 36].

Airway area measurements in the present study demonstrated a significant augmentation at the NPAA and OPAA levels following correction of the maxillary incisors’ axial inclination, whereas the change observed at the LPAA level was not statistically significant. These findings corroborate the results of Marzouk and El Kalza [23], who reported similar significant increases in airway dimensions after correction of the retroclined upper incisors and consequent overjet establishment. The observed airway augmentation is plausibly linked to the functional anterior shift of the mandible, occurring after the elimination of the occlusal interference from the retroclined upper incisors [37]. This interpretation is advocated by the significant skeletal findings reported in the current study; namely, a noteworthy increase in the SNBº angle, and a concurrent decrease in the FMAº, confirming the forward and upward positioning of the mandible following treatment. Furthermore, Uslu-Akcam [38] has demonstrated stasis in nasopharyngeal and oropharyngeal growth during the post-peak growth period, which aligns with the age range of the current study’s participants. This observation eliminates the confounding effect of growth on airway dimension changes. Consequently, any observed increase in airway space coincides with anterior mandibular repositioning achieved through deep bite correction.

Dentally, the significant labial tipping of maxillary incisors (mean U1-FH° increase of 17.36º) resulted from progressive wire sequencing up to 0.019 × 0.025-in, allowing full torque expression. This proclination is a vital therapeutic maneuver in Class II/2 management as it “unlocks” the mandible by eliminating the mechanical interference of retroclined incisors. By establishing a favorable overjet, this dental change acted as a catalyst for functional forward mandibular displacement; evidenced by increased SNB° and Ba-Ar distance, which eventually expanded the nasopharyngeal and oropharyngeal airway spaces. These findings align with similar axial inclination improvements (approximately + 15.2º) reported in previous Class II/2 investigations [39].

The implemented treatment induced significant alterations in the sagittal skeletal dimensions. A notable SNAº increase was observed, with a 1.06 º ± 0.49 change primarily ascribed to remodelling of the basal bone surrounding point A after the correction of the maxillary incisors’ inclination. This finding is consistent with prior studies, such as that by Shaik et al. [22], which utilized similar age- and growth-matched cohorts.

Moreover, in the present study, a simultaneous increase in the SNBº angle contributed to a significant decrease in the ANBº values, indicating a positive net change in the intermaxillary relationship with a mesial mandibular base shift, as priorly highlighted. These outcomes are in tune with the findings of a myriad of studies investigating Class II/2 cases, and the associated distal mandibular lock with the relative posterior condylar displacement. These include Gong et al. [40] and Fathallah et al. [41], where both investigations confirmed the anterior and inferior movement of mandibular condyles after proclination of maxillary incisors and bite opening in Class II/2 subjects. Conversely, prior studies report findings that conflict with the present results. Pullinger et al. [42] observed no significant correlation between condylar position and incisal overjet or overbite. Similarly, the study by Demisch et al. [43] failed to validate spontaneous anterior mandibular repositioning following the proclination of maxillary incisors. This discrepancy may be attributable to the use of appliances incorporating anterior bite plates; such designs often induce a downward and backward mandibular rotation, potentially neutralizing any measurable forward repositioning effect.

On the same note, and as per the increase in the Ba-Ar distance documented in the present study, a forward mandibular shift has been promoted after the implemented treatment phase; an interpretation that is advocated by the significant SNB° and U1-FH° increases, as well as the significant ANB° decrease. This pertains to the fact that the Ba-Ar distance is contemplated as a reliable constant in cephalometric growth analysis. According to Ricketts [44], Basion typically drifts posteriorly at a rate of 1.0 mm/year; Articulare mirrors this movement, maintaining a stable relationship between the two points. Coben [45] further confirmed this by identifying the Articulare point as a stable landmark. Because this baseline is predictable, an increase in the Ba-Ar distance may be suggestive of forward mandibular displacement, assuming the clinician has ruled out postural protrusion during imaging. In contrast to our results, Al-Nimri et al. [46] observed no statistically significant alterations in the Ba-Ar distance within their sample of treated Class II, Division 2 cases.

In summary, a primary concern in studies involving adolescent subjects is the confounding effect of natural craniofacial growth; hence, participants in this trial were specifically recruited in the decelerating stage of the adolescent growth spurt (CVMI stages 4 and 5). With significant nasopharyngeal growth deceleration instigated around the age of 13, which falls within the recruited age range in the present study, in addition to the relatively short treatment interval for the levelling and alignment phase (6 months), the statistically significant increases in the NPAA and OPAA areas are more likely attributable to the functional anterior shift of the mandible than to chronological growth. This interpretation is further supported by the predictable spatial Ba-Ar relationship during growth, thus the documented significant increase in the Ba-Ar distance may indicate forward functional mandibular displacement following the ‘unlocking’ of the retroclined maxillary incisors.

A significant reduction in vertical dimension was also observed following initial maxillary teeth alignment, occurring alongside the aforementioned forward mandibular displacement. This could be explained by the fact that levelling of the maxillary arch through relative incisor proclination and posterior extrusion initially ‘unlocked’ the mandible, facilitating a transition from a forced retrusive position to a more favorable functional posture. The observed vertical reduction following this treatment phase in Class II/2 patients has also been attributed by many investigators to the high-force neuromuscular environment and the ‘mandibular cinch’ effect of the masseter muscles, which resists permanent posterior extrusion and makes the correction of a deep bite a rather arduous task, especially in subjects with strong musculature [2, 5, 47].

### Clinical relevance

The clinical significance of these findings lies in the identification of a functional respiratory benefit that may be achieved during the very first stage of Class II/2 orthodontic correction. By labially tipping the retroclined maxillary incisors, the mechanical “lock” on the mandible is removed, facilitating a spontaneous forward functional shift. This repositioning is not merely a skeletal change, but a vital clinical maneuver that facilitates the augmentation of the nasopharyngeal and oropharyngeal airway areas. These results suggest that for late adolescent patients, the levelling and alignment phase serves as a critical window to enhance upper airway patency and establish a more favorable physiological environment for subsequent orthodontic mechanics. However, further research with a control group is needed to isolate the rendered treatment effect from residual growth.

### Limitations

A primary limitation of this study is the absence of a parallel, untreated Class II/2 control group. While each patient served as their own control to evaluate immediate functional responses (T1 vs. T0), the lack of a separate cohort limits the ability to definitively isolate orthodontic effects from natural craniofacial growth. However, withholding treatment from symptomatic Class II/2 patients to form an untreated control group presents significant ethical challenges in a clinical setting. To mitigate this, participants in the CVMI stages 4 and 5 were recruited, where nasopharyngeal growth naturally slows. However, residual growth over the six-month period cannot be entirely excluded. Consequently, the observed SPAD increase should be interpreted as strongly associated with, rather than exclusively caused by, the levelling and alignment phase. Second, the utilization of lateral cephalometric radiographs for airway assessment. Although these are cost-effective and show a high correlation with volumetric data, they remain a 2D representation of complex 3D structures, which may not capture lateral airway changes. Moreover, the outcomes were measured immediately following the levelling and alignment stage (approximately six months). Consequently, the long-term stability of the SPAD and mandibular position remains unknown. Furthermore, sample demographics are to be considered, since the study focused specifically on Class II/2 patients in the decelerating growth stage. Therefore, these results may not be generalizable to adult patients or those in earlier stages of development. Finally, the use of the Ba-Ar distance as a metric for mandibular advancement has inherent limitations, as it is a 2D representation of a complex 3D movement. While it serves as a valuable clinical indicator in this study, future research incorporating 3D condylar tracking could provide a more definitive analysis of functional displacement.

## Conclusions

The levelling and alignment phase of maxillary arch treatment in Class II division 2 patients during the decelerating growth stage suggest a significant association with skeletal, dental, and airway improvements. By “unlocking” the mandible through the correction of retroclined maxillary incisors, the following were observed:

- Airway Augmentation: A significant increase in the sagittal pharyngeal airway dimension at the nasopharyngeal (NPAA) and oropharyngeal (OPAA) levels. While natural growth cannot be entirely excluded, the rapid change suggests that these improvements are closely linked to the functional mandibular response.
- Mandibular Repositioning: Skeletal changes consistent with forward mandibular repositioning, evidenced by a significant increase in the SNB° angle, and a corresponding increase in the Ba-Ar distance.
- Dental Correction: Significant labial tipping of the maxillary incisors (U1-FH°), which “unlocks” the mandible and facilitates its anterior shift.
- Skeletal Harmony: The treatment results in a significant reduction in the ANB° angle, indicating an improved intermaxillary relationship, along with a significant reduction in the vertical dimension (FMA°).

Ultimately, these findings suggest that establishing proper incisor inclination and overjet in Class II/2 patients can facilitate a functional anterior shift of the mandible, thereby expanding the upper pharyngeal airway space.

## Data Availability

The datasets used and/or analyzed during the current study are available from the corresponding author on reasonable request.

## Abbreviations

Class II/2: Class II division 2
SPAD: Sagittal pharyngeal airway dimension
CVMI: Cervical vertebral maturational index
TMJ: Temporomandibular joint
FOV: Field of view
NPAA: Nasopharyngeal airway area
OPAA: Oropharyngeal airway area
LPAA: Laryngopharyngeal airway area
NHP: Natural head position
H: Harmonium
PNS: Posterior nasal spine
FH: Frankfurt horizontal plane
Ba-Ar: Basion-Articulare
C5AI: Antero-inferior point of the 5th cervical vertebra
FMA: Frankfurt-mandibular plane angle
ICC: Intraclass correlation coefficient
3D: Three-dimensional
CBCT: Cone-beam computed tomography

## References

[1] Bishara SE (ed) (2006) editor Class II malocclusions: diagnostic and clinical considerations with and without treatment. Semin Orthod

[2] Mahmoud YM, Samsudin AR, Al-Bayatti S, Pattanaik S, Gaballah K, Badran S et al (2026) A Study on the Association between Skeletal Malocclusion, Upper Airway Cross-Sectional Area, and Upper Airway Volume Using CBCT Scans. Eur J Gen Dent 15(01):089–98

[3] Swann GC (1954) The diagnosis and interception of Class II, Division 2 malocclusion. Am J Orthod 40(5):325–340

[4] Erickson LP, Hunter WS, Class II (1985) Division 2 Peatment and Mandibular Growth. Angle Orthod 55(3):215–224 3863502 10.1043/0003-3219(1985)055<0215:CIDPAM>2.0.CO;2

[5] Nielsen IL (2021) Etiology, development, diagnosis and considerations in treatment of the Class II, Division 2 malocclusion: what the clinician should know about this malocclusion (Part I). Taiwan J Orthod 33(1):1

[6] Peck S, Peck L, Kataja M, Class II (1998) Division 2 malocclusion: a heritable pattern of small teeth in well-developed jaws. Angle Orthod 68(1):9–20 9503130 10.1043/0003-3219(1998)068<0009:CIDMAH>2.3.CO;2

[7] Jain N, Soni S (2021) An overview of class II division 2 malocclusion. Int J Health Sci 5:214–221

[8] Cleall JF, Begole EA (1982) Diagnosis and treatment of class II division 2 malocclusion. Angle Orthod 52(1):38–60 6950679 10.1043/0003-3219(1982)052<0038:DATOCI>2.0.CO;2

[9] Ricketts RM (1950) Variations of the temporomandibular joint as revealed by cephalometric laminagraphy. Am J Orthod 36(12):877–898 14783193 10.1016/0002-9416(50)90055-8

[10] Gupta SP (2018) Functional Shift During Orthodontic Correction of Class II Division 2 Malocclusion in an Adult- A Rare Case Report. Archives Dentistry Oral Health 1(1):22–28

[11] Akin M, Ucar FI, Chousein C, Sari Z (2015) Effects of chincup or facemask therapies on the orofacial airway and hyoid position in Class III subjects. J Orofac Orthop 76(6):520–530 10.1007/s00056-015-0315-3 26446505

[12] Aboulfotouh M, Attia K, ElFeky H (2021) Three-dimensional effects of maxillary protraction on Pharyngeal Airway. Egypt Dent J 67:71–78

[13] Oktay H, Ulukaya E (2008) Maxillary protraction appliance effect on the size of the upper airway passage. Angle Orthod 78(2):209–214 18251620 10.2319/122806-535.1

[14] El H, Palomo JM (2011) Airway volume for different dentofacial skeletal patterns. Am J Orthod Dentofac Orthop 139(6):e511–e521 10.1016/j.ajodo.2011.02.015 21640863

[15] El H, Palomo JM (2013) An airway study of different maxillary and mandibular sagittal positions. Eur J Orthod 35(2):262–270 22045695 10.1093/ejo/cjr114

[16] Kaygisiz E, Tuncer BB, Yüksel S, Tuncer C, Yildiz C (2009) Effects of maxillary protraction and fixed appliance therapy on the pharyngeal airway. Angle Orthod 79(4):660–667 19537871 10.2319/072408-391.1

[17] Lee JW, Park KH, Kim SH, Park YG, Kim SJ (2011) Correlation between skeletal changes by maxillary protraction and upper airway dimensions. Angle Orthod 81(3):426–432 21299388 10.2319/082610-499.1 PMC8923552

[18] Hong JS, Oh KM, Kim BR, Kim YJ, Park YH (2011) Three-dimensional analysis of pharyngeal airway volume in adults with anterior position of the mandible. Am J Orthod Dentofac Orthop 140(4):e161–e169 10.1016/j.ajodo.2011.04.020 21967954

[19] Xu J, Sun R, Wang L, Hu X (2019) Cone-beam evaluation of pharyngeal airway space in adult skeletal Class II patients with different condylar positions. Angle Orthod 89(2):312–316 30457352 10.2319/040518-253.1 PMC8120870

[20] Institution BS (1983) British standard glossary of dental terms. BSI, London

[21] Kazem A-N, Abo-Zomor M, Alomari S (2016) Changes in mandibular position in treated Class II division 2 malocclusions in growing and non-growing subjects. Aust Orthod J 32(1):73–81 27468594

[22] Shaik S, Reddy GV, Perala J, Thejasri K, Singaraju GS, Mandava P et al (2022) Sagittal positional changes of the mandible following alignment and levelling of class ii division 2 cases: An observational study in decelerating stages of adolescent growth spurt. Cureus 14(12):e32653 10.7759/cureus.32653 PMC9845512 36660498

[23] Marzouk E, Kalza EL (2014) Three dimensional evaluation of pharyngeal airway changes associated with maxillary incisors proclination in class II division 2 using cone-beam computed tomography. Egypt Orthod J 45:1–18

[24] Petrie A, Sabin C (2019) Medical statistics at a glance, 3rd edn. Wiley, West Sussex, UK

[25] Ramirez-Yañez G, Sidlauskas A, Junior E, Fluter J (2007) Dimensional changes in dental arches after treatment with a prefabricated functional appliance. J Clin Pediatr Dent 31(4):279–283 19161066 10.17796/jcpd.31.4.d7p31201572n72h2

[26] Chand K, Jacob S, Charles A (2017) Assesment of changes in the sagittal pharyngeal airway dimensions post twin-block therapy using polar planimeter. SRM J Res Dent Sci 8(2):51–57

[27] Koo TK, Li MY (2016) A Guideline of Selecting and Reporting Intraclass Correlation Coefficients for Reliability Research. J Chiropr Med 15(2):155–163 27330520 10.1016/j.jcm.2016.02.012 PMC4913118

[28] Tourne LP (1991) Growth of the pharynx and its physiologic implications. Am J Orthod Dentofac Orthop 99(2):129–139 10.1016/0889-5406(91)70115-D 1990822

[29] Jeans W, Fernando D, Maw A, Leighton B (1981) A longitudinal study of the growth of the nasopharynx and its contents in normal children. Br J Radiol 54(638):117–121 7459548 10.1259/0007-1285-54-638-117

[30] Kim JE, Yim S, Choi JY, Kim S, Kim SJ, Baek SH (2020) Effects of the long-term use of maxillary protraction facemasks with skeletal anchorage on pharyngeal airway dimensions in growing patients with cleft lip and palate. Korean J Orthod 50(4):238–248 32632043 10.4041/kjod.2020.50.4.238 PMC7369382

[31] Elfouly D, Dumu EJ, Madian AM, Eid FY (2024) The effect of different functional appliances on the sagittal pharyngeal airway dimension in skeletal class II: a retrospective study. Sci Rep 14(1):19410 39169053 10.1038/s41598-024-69717-5 PMC11339275

[32] Eid FY, Abbas BA, Elfouly DA, Madian AM (2024) A retrospective study evaluating the influence of Class III correction appliances on the sagittal pharyngeal airway dimension. Sci Rep 14(1):7340 38538631 10.1038/s41598-024-57614-w PMC10973336

[33] Restrepo C, Santamaría A, Peláez S, Tapias A (2011) Oropharyngeal airway dimensions after treatment with functional appliances in class II retrognathic children. J Oral Rehabil 38(8):588–594 21294763 10.1111/j.1365-2842.2011.02199.x

[34] Riley R, Powell N, Guilleminault C (1986) Cephalometric roentgenograms and computerized tomographic scans in obstructive sleep apnea. Sleep 9(4):514–515 3809865 10.1093/sleep/9.4.514

[35] Aboudara C, Nielsen I, Huang JC, Maki K, Miller AJ, Hatcher D (2009) Comparison of airway space with conventional lateral headfilms and 3-dimensional reconstruction from cone-beam computed tomography. Am J Orthod Dentofac Orthop 135(4):468–479 10.1016/j.ajodo.2007.04.043 19361733

[36] Madian AM, Elfouly D (2023) Cephalometric changes in pharyngeal airway dimensions after functional treatment with twin block versus myobrace appliances in developing skeletal class II patients: a randomized clinical trial. BMC Oral Health 23(1):998 38093237 10.1186/s12903-023-03701-9 PMC10720117

[37] Rodrigues J, Samsudin AR, Ismail A, Bayatti SA, Pattanaik S, Kamath V et al (2025) Cephalometric Evaluation of Pharyngeal Airway Space among Different Skeletal Malocclusions in United Arab Emirates Residents: A Cross-Sectional Study. Pesqui Bras Odontopediatria Clín Integr 25:230173

[38] Uslu-Akcam O (2017) Pharyngeal airway dimensions in skeletal class II: A cephalometric growth study. Imaging Sci Dent 47(1):1–9 28361023 10.5624/isd.2017.47.1.1 PMC5370247

[39] Devreese H, De Pauw G, Van Maele G, Kuijpers-Jagtman A, Dermaut L (2007) Stability of upper incisor inclination changes in Class II division 2 patients. Eur J Orthod 29(3):314–320 17483493 10.1093/ejo/cjm011

[40] Gong F, Tao L, Cao H (2003) A clinical study of the changes of condylar position in class division 2 deep-bite patients after orthodontic treatment. Shanghai J Stomatol 12(5):334–337 14966605

[41] Fathallah M, El-Kenany W, Ismail H, Mowafy MI (2012) Three-dimensional evaluation of possible mandibular functional displacement in class II division 2 malocclusion subjects. Egypt Orthod J 42:15–32

[42] Pullinger AG, Solberg WK, Hollender L, Petersson A (1987) Relationship of mandibular condylar position to dental occlusion factors in an asymptomatic population. Am J Orthod Dentofac Orthop 91(3):200–206 10.1016/0889-5406(87)90447-1 3469906

[43] Demisch A, Ingervall B, Thüer U (1992) Mandibular displacement in Angle Class II, division 2 malocclusion. Am J Orthod Dentofac Orthop 102(6):509–518 10.1016/0889-5406(92)70067-K 1456231

[44] Ricketts RM (1979) Bioprogressive therapy. Denver

[45] Coben SE (1955) The integration of facial skeletal variants: a serial cephalometric roentgenographic analysis of craniofacial form and growth. Am J Orthod 41(6):407–434

[46] Al-Nimri K, Abo-Zomor M, Alomari S (2016) Changes in mandibular position in treated Class II division 2 malocclusions in growing and non-growing subjects. Aust Orthod J 32(1):73–81 27468594

[47] Tentolouri E, Antonarakis GS, Georgiakaki I, Kiliaridis S (2022) Masseter muscle thickness and vertical cephalometric characteristics in children with Class II malocclusion. Clin Exp Dent Res 8(3):729–736 35150084 10.1002/cre2.528 PMC9209807

